# Cholecalciferol vs. calcifediol supplementation on visceral adiposity in people with obesity: a real-world retrospective study

**DOI:** 10.3389/fnut.2025.1676668

**Published:** 2025-11-19

**Authors:** Martina Chiurazzi, Mariana Di Lorenzo, Maria Serena Lonardo, Mariastella Di Lauro, Carmela Onda, Concetta Sozio, Daniela Pacella, Nunzia Cacciapuoti, Bruna Guida, Ciro Menale

**Affiliations:** 1 Physiology Nutrition Unit, Department of Clinical Medicine and Surgery, University of Naples Federico II, Napoli, Italy; 2 Department of Public Health, University of Naples Federico II, Napoli, Italy

**Keywords:** vitamin D deficiency, cholecalciferol, calcifediol, obesity, adiposity

## Abstract

**Introduction:**

Nowadays it is well known that obesity and vitamin D deficiency are closely linked. In this view, this study aimed to assess the effects of two different Vitamin D formulations, combined with a Mediterranean hypocaloric diet (MHD) on 25(OH)D concentration, weight loss and visceral adiposity in subjects with obesity and vitamin D insufficiency/deficiency.

**Methods:**

Eighty-four patients with obesity were retrospectively selected and divided into three groups according to the type of treatment received: MHD alone (C group), MHD + cholecalciferol (D group), and MHD + calcifediol (N group). 25(OH)D concentration, anthropometric parameters, body composition and visceral adiposity indices (LAP, VAI, NVAI) were assessed at baseline and after 3 months.

**Results:**

All groups showed significant reductions in anthropometric parameters after 3 months. Notably, Group N achieved the greatest increase in serum 25(OH)D (+20 ng/mL), the highest weight loss (−7.8 Kg) and a significant improvement in LAP and NVAI. In addition, only N group showed an increased fat-free mass. Regression analysis confirmed a significant association between calcifediol treatment and LAP reduction, independent of BMI.

**Discussion:**

Calcifediol supplementation, combined with a MHD, is more effective than cholecalciferol in improving vitamin D status and reducing visceral adiposity in subjects with obesity. These findings support the recommended use of calcifediol supplementation in obesity-related vitamin D deficiency management.

## 1 Introduction

Obesity is a chronic, multifactorial condition marked by excessive fat accumulation due to an imbalance between caloric intake and energy expenditure. It is a significant risk factor for metabolic, cardiovascular, respiratory, musculoskeletal, and oncological diseases, contributing to increased morbidity and mortality while reducing quality of life. The rising global prevalence of obesity has made it a pressing public health concern (1, 2). Among its various health consequences, obesity is closely linked to vitamin D deficiency, although the exact mechanisms remain under investigation (3). Epidemiological and clinical studies have consistently shown an inverse association between central adiposity and circulating 25-hydroxyvitamin D (25(OH)D) levels. Low vitamin D concentrations have been observed in patients with obesity across diverse populations, independent of ethnicity or geography. Severe deficiency in this group has been further associated with heightened risks of cardiovascular disease, respiratory conditions, osteoporosis, and cancer (4, 5). A 2023 study highlights the role of visceral adipose tissue (VAT) in vitamin D homeostasis, suggesting that stored vitamin D may become sequestered in excess fat, reducing its bioavailability. Even with cholecalciferol supplementation, individuals with high VAT often hardly reach normal 25(OH)D concentration, implying impaired release or utilization, possibly due to sequestration of vitamin D in visceral fat and altered metabolic processing. These findings emphasize the importance of reducing visceral fat through lifestyle modifications to improve vitamin D status (6).

Vitamin D plays a crucial role in bone health and mineral balance, particularly in calcium and phosphorus regulation. Although the optimal plasma 25(OH)D concentration remains debated, deficiency is generally defined as <20 ng/mL, insufficiency as 20–30 ng/mL, and sufficiency as >30 ng/mL, with some guidelines suggesting an optimal range of 40–60 ng/mL for maximal health benefits (7–9). The primary causes of deficiency include inadequate sun exposure, impaired skin synthesis, and sequestration in adipose tissue. Additionally, the dilution effect from excess body fat may further lower circulating 25(OH)D concentration (8). Several therapeutic options exist for vitamin D deficiency, with cholecalciferol (vitamin D₃) and calcifediol (25-hydroxyvitamin D₃) being the most commonly used. While cholecalciferol must first be converted in the liver to calcifediol, the latter is already an active metabolite, requiring fewer metabolic steps to exert its effects (10). Despite these differences, cholecalciferol remains widely used in the treatment of vitamin D deficiency, primarily due to its availability, lower cost, and established clinical experience.

Given the variations in the metabolism of different forms of vitamin D, studies suggest that calcifediol is more effective in rapidly increasing serum 25(OH)D concentration compared to cholecalciferol, making it a potentially superior option, particularly for patients with obesity who may experience altered vitamin D metabolism (10). However, it remains unclear whether the observed improvement in vitamin D status among patients with obesity treated with both vitamin D supplementation and a hypocaloric diet is primarily attributable to the supplementation itself or to weight loss also. Some studies have reported that weight loss alone may increase circulating 25-hydroxyvitamin D [25(OH)D] levels, likely due to reduced sequestration in adipose tissue (11). In addition, studies suggest that vitamin D supplementation, when combined with a low-calorie diet, may enhance weight reduction beyond that achieved by dietary intervention alone (12), indicating a potential active role of vitamin D in weight regulation. Furthermore, the majority of available studies have used cholecalciferol rather than calcifediol formulations, limiting the applicability of these findings to other forms of supplementation. This highlights the need for further research to clarify the direction and mechanisms underlying this relationship. Therefore, this study aimed at evaluating the effects of Vitamin D supplementation in combination with a low-calorie Mediterranean diet on serum 25(OH)D concentration and weight loss in patients with obesity characterized by Vitamin D insufficiency or deficiency, comparing two different formulations and focusing on visceral adiposity parameters.

## 2 Methods

### 2.1 Study design

The study protocol was approved by the Ethical Committee of the “Federico II” University Medical School of Naples (EC approval code: [309/22]), and all participants provided written informed consent.

A group of 82 patients with obesity (BMI ≥ 30 kg/m^2^) attending the Outpatient Clinic of the Departmental Program “Diet Therapy in Transplantation and Chronic Renal Failure” at the School of Medicine, University of Naples “Federico II” was retrospectively selected from 2017 to 2021. Based on the analysis of the medical records, 59 subjects within this group were identified as having Vitamin D insufficiency or deficiency [25(OH)D plasma levels <20 ng/mL]. All patients received Mediterranean Hypocaloric Diet (MHD) treatment to achieve weight loss, and patients with vitamin D insufficiency or deficiency underwent Vitamin D supplementation as described below.

At baseline and after 3 months, demographic and clinical characteristics, biochemical parameters, pharmacological treatments, anthropometric measurements, body composition, and physical activity data were collected. Following data analysis, it was found that the 59 eligible subjects had adhered to both the MHD and the prescribed vitamin D supplementation, while the remaining participants adhered to the prescribed diet regimen alone. Therefore, the subjects were divided into three groups according to the type of supplement they had been prescribed, basing on good clinical practice standards: the first group (C group, *n* = 23) adhering to the MHD without vitamin D supplementation; the second group (D group, *n* = 24) following the MHD and receiving cholecalciferol supplementation; the third group (N group, *n* = 35) following a MHD combined with calcifediol supplementation. Participants in the D group received a monthly dose of 25,000 IU of cholecalciferol for 3 months, while those in the N group were treated with 0.266 mg/monthly of calcifediol for the same duration. Cholecalciferol 25,000 IU was prescribed as a single monthly dose, consistent with the reference treatment defined in the guidelines (cholecalciferol 800 IU/day ≈ 25,000 IU/month, or 0.625 mg/month). The monthly administration of 0.266 mg of calcifediol was selected because, for long-term treatment, this dosage has been shown to produce stable and sustained 25(OH)D concentrations (13–16). Monthly supplementation was also chosen to ensure better adherence compared to daily or weekly administration. Exclusion criteria were the presence of conditions known to interfere with vitamin D metabolism or absorption, such as intestinal malabsorption syndromes, chronic kidney or liver disease, cancer, type 1 or 2 diabetes, and thyroid disorders. The final sample size was determined by the number of eligible patients identified within the predefined time frame as described in statistical analysis section.

### 2.2 Study protocol

Participants were assessed both at baseline (T0) and after 3 months of treatment (T3) following standardized protocols. To evaluate their nutritional status, anthropometric measurements were taken, including weight (measured using a Seca GmbH & Co KG scale, Hamburg, Germany), height (using a wall-mounted stadiometer with precision to the nearest 0.1 cm), body mass index (BMI), waist circumference (WC), and hip circumference (17). In order to assess body composition, bioelectrical impedance analysis (BIA) was conducted using a tetrapolar device (RJL 101; Akern SRL, Florence, Italy) with a single-frequency measurement (50 kHz) (18). Additionally, several blood parameters were measured throughout the treatment period, including blood glucose (Glucose), insulin, total cholesterol (TC), HDL cholesterol (HDL-C), LDL cholesterol (LDL-C), triglycerides (TG), Calcium and 25(OH)D concentration. These represent the main nutritional and metabolic assessment parameters used in clinical practice. Among these, parameters necessary for the calculation of adiposity indices, that we have considered, are present. In fact, to better estimate visceral fat distribution and dysfunction, which are associated with metabolic risks, specific adiposity indices were calculated by combining anthropometric measures (such as waist circumference) with metabolic markers (such as lipid levels), serving as surrogate indicators of visceral adiposity and cardiometabolic risk. These indices are particularly useful for assessing the distribution of body fat, especially around the abdominal region, where visceral fat tends to accumulate. Research has shown that low levels of vitamin D are linked to increased visceral adiposity, which may contribute to metabolic disorders such as insulin resistance and cardiovascular disease (19). The following adiposity indices were used to evaluate fat distribution:- Visceral Adiposity Index (VAI): This complex index takes into account waist circumference, BMI, triglyceride (TG) levels, and HDL cholesterol (HDL-C) levels. The formula varies for men and women:


$$\begin{array}{l} \text{For}\;\mathrm{men}:\mathrm{VAI}=(\mathrm{WC})/[39.68+(1.88\times \mathrm{BMI})]\times \\ \left(\mathrm{TG}/1.03)\times (1.31/\mathrm{HDL}\right) \end{array}$$

$$\begin{array}{l} \text{For women}:\mathrm{VAI}=(\mathrm{WC})/[36.58+(1.89\times \mathrm{BMI})]\times \\ \left(\mathrm{TG}/0.81)\times (1.52/\mathrm{HDL}\right) \end{array}$$

- Lipid Accumulation Product (LAP): This index uses waist circumference and triglyceride levels to further evaluate fat distribution (20):


Formen:LAP=(WC−65)×TG

For women:LAP=(WC−58)×TG

- The new visceral adiposity index (NVAI): This index uses mean arterial pressure (MAP), waist circumference (WC), triglyceride (TG) and HDL cholesterol (HDL) levels (21).


$$\text{For}\;\mathrm{men}:\text{NVAI}=1/[1+\exp\;\{–(\begin{array}{l} –21.858+(0.099\times \mathrm{age})+\\ (0.10\times \mathrm{WC})+(0.12\times \mathrm{MBP})+\\ \left(0.006\times \mathrm{TG})+(\begin{array}{l} –0.077\times \\ \mathrm{HDL} \end{array}\right) \end{array})\}]$$

$$\text{For women}:\text{NVAI}=1/[1+\exp\{–(\begin{array}{l} –18.765+(0.058\times \mathrm{age})+\\ (0.14\times \mathrm{WC})+(\begin{array}{l} 0.057\times \\ \mathrm{MBP} \end{array})+\\ \left(0.004\times \mathrm{TG})+(\begin{array}{l} –0.057\times \\ \mathrm{HDL} \end{array}\right) \end{array})\}]$$

These adiposity composite indices offer a more detailed and precise way to assess visceral fat, which is crucial given its association with low 25(OH)D concentration and its potential role in metabolic dysfunction. Understanding visceral fat distribution can provide valuable insights into metabolic health and help identify patients at higher risk for conditions related to both obesity and vitamin D deficiency.

### 2.3 Dietary treatment and compliance

A personalized diet was tailored for each patient in all groups, following the guidelines set by the LARN (Livelli di Assunzione Raccomandata di Nutrienti) (22). All participants were recommended to follow a Mediterranean hypocaloric diet, with a caloric intake reduced by approximately 20–30% relative to their estimated daily energy requirements. Energy needs were calculated based on basal metabolic rate (BMR), estimated using the revised Harris-Benedict equation, and adjusted according to physical activity level, as per international guidelines (23).

Dietary adherence was monitored monthly through structured interviews conducted by dietitians using Food Frequency Questionnaires (FFQs). Nutrient and energy intake were calculated by comparing FFQ responses with standardized food composition tables (24).

### 2.4 Statistical analysis

A *post hoc* power analysis was performed using the observed effect size (Cohen’s *d* = 0.78) calculated from the difference in body weight variation between the control group C (*n* = 23, *Δ* = 3.2 ± 5.7 kg) and the treatment group N (*n* = 35, Δ = 7.9 ± 6.3 kg). With an alpha error of 0.05, the achieved statistical power (1–*β* error) was 0.81, indicating that the sample size was sufficient to detect a significant difference between groups. Categorical variables are presented as absolute numbers and percentages (%). The Kolmogorov–Smirnov test was used to assess the normality of data distribution. Normally distributed variables are expressed as the mean ± standard deviation (SD), while non-normally distributed variables are reported as the median and interquartile range (IQR). Within-group comparisons between baseline and follow-up were performed using a paired t-test. Comparisons between independent groups were conducted using one-way ANOVA. Bonferroni correction was used for post-hoc pairwise comparisons (t(cp)= corrected Paired Student’s t-test). A mixed-effects linear regression model was applied to assess the association of covariates (Vitamin D treatment, BMI and WC) with changes in LAP and NVAI after 3 months using time and ID as random effects. We limited our predictors (namely, BMI and WC) to those with established associations with LAP and NVAI. All variables included in the regression model had a gVIF ≤ 5. Correlation was calculated using Pearson’s correlation coefficient. No missing data were present for the variables included in the analyses; therefore, no imputation procedures were applied. Statistical analyses were performed using SPSS version 20.0 (SPSS Inc., Chicago, IL, United States), GraphPad Prism 9.0 (GraphPad Software, Inc., La Jolla, CA, United States) or R statistical software (version 4.4.0), with statistical significance set at *p* < 0.05.

## 3 Results

### 3.1 Baseline characteristics

Baseline demographic characteristics, anthropometric measurements and major comorbidities of the study population (*n* = 82, 29.3% male; mean age: 44 ± 12.2 years; mean BMI: 39.7 ± 9.4 kg/m^2^), divided into three groups, are summarized in Table 1. No significant differences were observed among the three groups at baseline in terms of anthropometric, demographic characteristics as well as major comorbidities. As expected, baseline 25(OH)D concentration were significantly lower in the N and D groups compared to C group [*F*(df = 81) = 31.1, *p* = 0.000], in line with their classification based on vitamin D insufficiency or deficiency identified through retrospective record analysis (Table 1).

**Table 1:** Baseline anthropometric, demographic characteristics, biochemical parameters and major comorbidities of the study population.

	C Group (n.23)	D Group (n.24)	N Group (n.35)
Male sex, *n* (%)	6 (26.1%)	6 (25.0%)	12 (34.3%)
Age, years	48.6 ± 12.8	42.9 ± 11.6	41.7 ± 11.7
BMI, Kg/m^2^	36.7 ± 8.2	39.9 ± 10.5	42.5 ± 8.8
Weight, Kg	98.5 ± 26.7	102.9 ± 30.6	113.9 ± 24.3
WC, cm	107.3 ± 18.7	109.4 ± 16.5	114.8 ± 15.5
Dyslipidemia (*n*,%)	9 (39.1%)	6 (25.0%)	11 (31.4%)
Hypertension (*n*,%)	12 (52.2%)	8 (33.3%)	9 (25.7%)
25(OH)D, ng/mL	25.3 ± 4.5	17.1 ± 5.8*	14.3 ± 4.9*

Continuous variables are expressed as mean ± SD, while categorical variables are presented as numbers and percentages. *p-values < 0.05 vs. C Group. Abbreviations: BMI, body mass index; WC, waist circumference; 25(OH)D, 25-hydroxy-vitamin D. Participants underwent monthly outpatient follow-ups, and the effects of different forms of vitamin D supplementation on the pre-specified parameters were evaluated after 3 months, yielding the following results.

### 3.2 Dietary patterns and effects of vitamin D supplementation on body composition and weight loss

**Figure 1:**
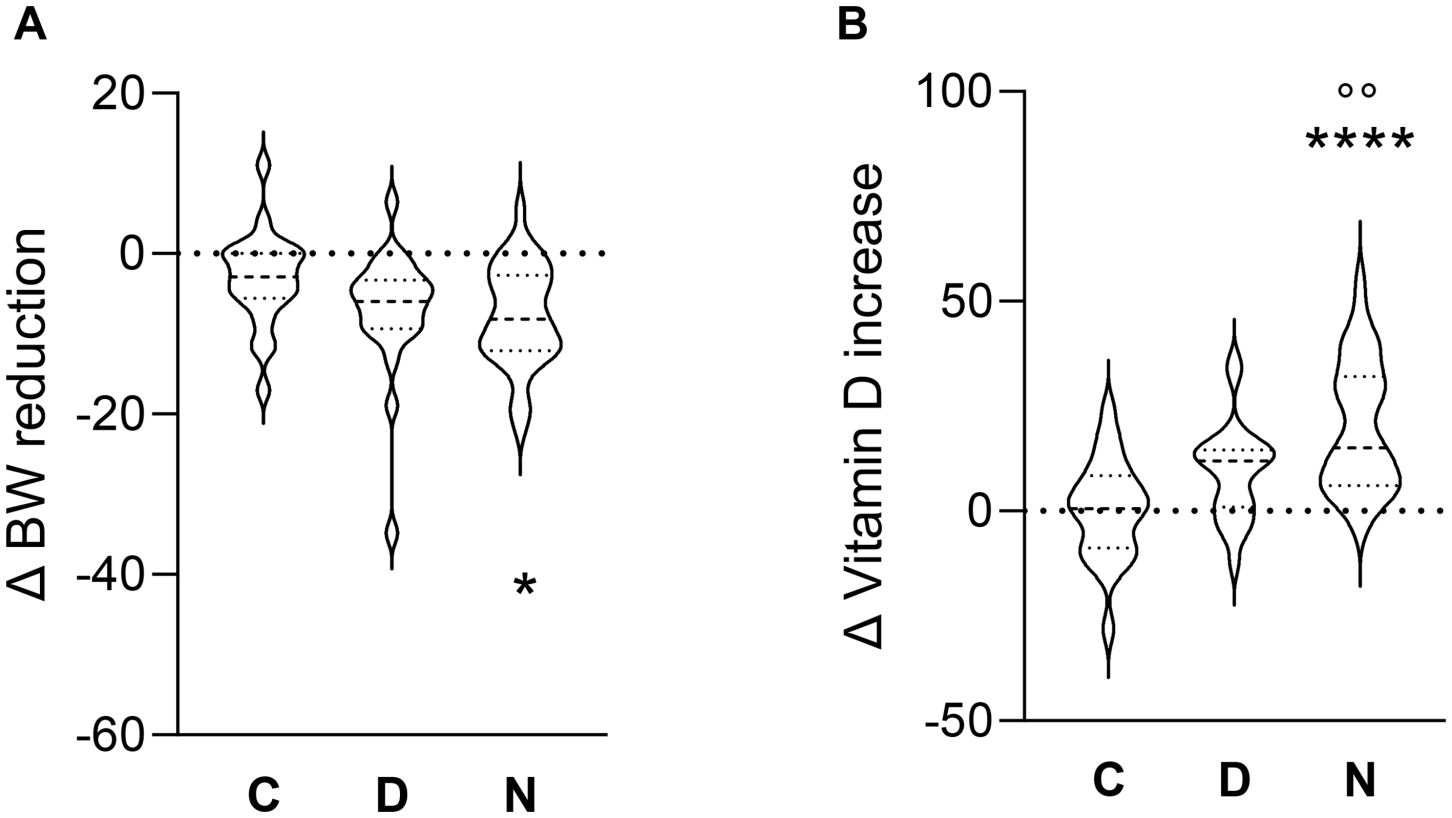
Violin plots showing distributions of the change (*Δ*) in body weight **(A)** and 25(OH)D concentration **(B)**, from baseline to 3 months, among the three groups. *p < 0.05, **** *p* < 0.0001, C vs N groups. °°*p* < 0.01 D vs N groups.

Table 2 presents the results after 3 months of treatments compared to baseline for each group. A significant reduction in body weight [t_(cp)_ = 2.6, *p* = 0.015, C group; t_(cp)_ = 4.5, *p* < 0.001, D group; *p* < 0.001, t_(cp)_ = 7.3, N group], BMI [t_(cp)_ = 2.6, *p* = 0.014, C group; t_(cp)_ = 3.6, *p* = 0.002, D group; t_(cp)_ = 4.9, *p* < 0.001, N group] and WC [t_(cp)_ = 3.9, *p* = 0.001, C group; t_(cp)_ = 2.8, *p* = 0.008, D group; t_(cp)_ = 4.6, *p* < 0.001, N group] was observed in all groups after 3 months. Furthermore, a significant reduction in percentage of fat mass was observed in both the D and N groups but not in the C group [t_(cp)_ = 2.3, *p* = 0.029, D group; t_(cp)_ = 2.6, *p* = 0.013, N group]. The N group showed also a significant improvement of free fat mass [t_(cp)_ = −2.5, *p* = 0.015]. Additionally, a significant reduction was observed in the biochemical parameter of TC [t_(cp)_ = 2.3, *p* = 0.031] in the D group only. On the other hand, a significant improvement in ferritin [t_(cp)_ = −2.1, *p* = 0.030] and uric acid [t_(cp)_ = 2.1, *p* = 0.046] was observed in the C group respect to D and N groups. Moreover, a significant improvement of albumin levels [t_(cp)_ = −4.1, *p* = 0.010] and of DSAP [t_(cp)_ = 2.3, *p* = 0.027] was observed in N group only. Our data indicated a significant improvement of levels of vitamin D [t_(cp)_ = −4.0, *p* = 0.001, D group; t_(cp)_ = −7.6, *p* < 0.001, N group] compared to their respective baseline, while the N group showed an increase in plasma 25(OH)D concentration also compared to C group. Calcium levels were also increased [t_(cp)_ = −2.5, *p* = 0.022] in N group only. To better define the effects of Vitamin D supplementation on BW and plasma 25(OH)D concentration, the differences at baseline (T0) and after 3 months (T3) in weight loss and Vitamin D increase were compared across the different groups (shown in Figures 1A,B). A decrease in body weight was observed [*F*(df = 81) = 3.7, *p* = 0.029, *Δ* Kg, −3.2, −7.1, −7.8, in the C, D, and N groups, respectively], with a significant weight loss in the N group compared to C group. A significant increase in 25(OH)D concentration was also observed [*F*(df = 81) = 15.5, *p* = 0.000, Δ ng/mL, +0.3, +9.3, +20.0, in the C, D, and N groups, respectively], with the N group showing higher increases compared to both the D and C groups.

**Table 2:** Anthropometric features, body composition characteristics, and metabolic parameters of the C, D, and N groups at baseline and after 3 months of treatment.

	C Group (n.23)		D Group (n.24)		N Group (n.35)	
	T0	T3	T0	T3	T0	T3
Weight, Kg	98.5 ± 26.7	95.3 ± 24.7*	102.9 ± 30.6	95.8 ± 28.0*	113.9 ± 24.3	106.0 ± 24.4*
BMI, kg/m^2^	36.7 ± 8.2	35.5 ± 7.4*	39.9 ± 10.5	36.8 ± 9.9*	42.5 ± 8.8	39.9 ± 8*
WC, cm	107.3 ± 18.7	102.9 ± 15.6*	109.4 ± 16.5	104.1 ± 16.4*	114.8 ± 15.5	109.7 ± 18.8*
FM, %	39.6 ± 7.8	37.7 ± 8.6	42.3 ± 9.8	39.7 ± 10.7*	43.0 ± 8.8	40.2 ± 10.3*
FFM,%	60.2 ± 7.8	62.3 ± 8.6	57.7 ± 9.8	59.1 ± 11.9	56.9 ± 8.8	59.8 ± 10.4*
TBW %	44.8 ± 5.7	46.4 ± 6.1*	52.9 ± 7.6	43.6 ± 8.7	41.7 ± 6.9	44.3 ± 6.8*
Phase angle, Φ	6.3 ± 1.2	5.9 ± 1.1	6.2 ± 1.0	6.1 ± 1.2	6.0 ± 1.4	6.2 ± 1.1
TC, mg/dL	180.9 ± 44.1	172.5 ± 45.2	198.3 ± 39.7	180.2 ± 37.2*	200.1 ± 40.5	187.5 ± 37.9
HDL-C, mg/dL	49.2 ± 9.6	49.2 ± 11.9	52.2 ± 12.4	51.2 ± 12.6	50.6 ± 13.0	49.1 ± 12.6
LDL-C, mg/dL	153.6 ± 38.6	147.3 ± 43.3	165.1 ± 34.6	148.7 ± 38.6	155.6 ± 40.4	165.4 ± 49.1
TG, mg/dL	103.7 ± 49.2	103.6 ± 56.4	113.4 ± 55.1	105.4 ± 34.8	175.5 ± 171.2	133.8 ± 70.9
Glucose, mg/dL	97.6 ± 17.3	94.6 ± 17.4	92.3 ± 10.4	89.3 ± 9.0	94.4 ± 22.6	95.7 ± 26.4
Hemoglobin, g/dL	13.5 ± 1.3	14.0 ± 2.0	12.1 ± 0.8	12.4 ± 0.9	13.0 ± 1.9	13.6 ± 0.7
Ferritin, ng/mL	7.2 ± 1.5	14.5 ± 2.0*	27.7 ± 20.8	37.8 ± 25.4	75.7 ± 49.4	88.3 ± 50.9
Uric Acid, mg/dL	5.5 ± 1.5	5.0 ± 1.1*	4.9 ± 1.8	4.7 ± 1.7	5.2 ± 1.0	5.2 ± 1.1
Albumin, g/dL	4.2 ± 0.2	4.0 ± 0.4	3.8 ± 0.5	3.9 ± 0.6	4.0 ± 0.2	4.3 ± 0.2*
DSAP, mmHg	80.5 ± 8.7	79.3 ± 9.2	78.9 ± 8.6	78.9 ± 9.1	82.6 ± 6.6	79.0 ± 9.4*
SBAP, mmHg	127.6 ± 12.6	125.3 ± 14.3	122.9 ± 12.7	121.6 ± 12.1	124.6 ± 10.4	121.2 ± 13.8
25(OH)D, ng/mL	25.3 ± 4.5	25.6 ± 10.6	17.1 ± 5.8	26.4 ± 9.7*	14.3 ± 4.9	34.4 ± 15.7*°
Phosphorus, mg/dL	3.6 ± 0.5	3.5 ± 0.7	3.3 ± 0.5	3.8 ± 0.1	3.4 ± 0.5	3.9 ± 0.6
Calcium, mg/dL	9.6 ± 0.6	9.8 ± 0.4	9.3 ± 0.6	9.3 ± 0.4	9.2 ± 0.5	9.4 ± 0.5*

Continuous variables are expressed as mean ± SD. *p < 0.05 vs. baseline; °*p* < 0.05 vs. C Group. BMI, body mass index; WC, waist circumference; FM, fat mass; FFM, fat-free mass; TBW, total body water; HDL, high-density lipoprotein; LDL-C, low-density lipoprotein cholesterol; TC, total cholesterol; TG, triglycerides; SBAP, systolic blood pressure; DBAP, diastolic blood pressure; 25(OH)D, 25-hydroxy-vitamin D.

### 3.3 Effects of vitamin D supplementation on visceral adiposity indices

To better characterize body adiposity distribution within the study population and to evaluate the effects of Vitamin D supplementation on adiposity, patients’ parameters were analyzed to obtain the adiposity indices: Visceral Adiposity Index (VAI), Lipid Accumulation Product (LAP), New Visceral Adiposity Index (NVAI), whose reduction indicates an improvement in adiposity parameter in patients with obesity. Table 3 shows a significant amelioration in the NVAI [t_(cp)_ = 2.3, *p* = 0.027] and LAP [t_(cp)_ = 2.2, *p* = 0.037] in the N group after 3 month of treatment compared to baseline, but no significant changes were observed in the C or D groups. Furthermore, our results indicate a negative correlation between baseline vitamin D levels and body weight (*r* = − 0.3; *p* = 0.003). To investigate a directional relationship between 25(OH)D concentration and the NVAI and LAP, and how plasma levels of Vitamin D after supplementation affect the indices as outcome, a mixed-effects linear regression model was performed (Table 4). This analysis revealed a significant association between LAP and vitamin D treatments with calcifediol compared to patients treated with cholecalciferol or without supplementation. A significant association was also reported between LAP and WC. However, BMI was not significantly associated with changes in LAP after 3 months of treatment. On the other hand, NVAI was not significantly associated with the different vitamin D treatments or BMI but showed a significant association with WC only.

**Table 3:** Visceral adiposity indices of the C, D, and N groups at baseline and after three months of treatment.

	C Group (n.23)		D Group (n.34)		N Group (n.35)	
	T0	T3	T0	T3	T0	T3
NVAI	0.7 ± 0.3	0.7 ± 0.4	0.7 ± 0.3	0.7 ± 0.3	0.8 ± 0.3	0.7 ± 0.3*
VAI	2.0 ± 1.2	2.0 ± 1.5	1.9 ± 1.1	1.8 ± 0.9	3.3 ± 4.1	2.4 ± 1.7
LAP	68.1 ± 44.9	61.6 ± 48.9	72.8 ± 39.6	62.3 ± 30.4	132.8 ± 144.2	93.9 ± 71.4*

Continuous variables are expressed as mean ± SD. *p < 0.05 vs. baseline. LAP, lipid accumulation product; VAI, visceral adiposity index; NVAI, new visceral adiposity index.

**Table 4:** Determinants of LAP and NVAI in study patients.

Outcome LAP (Model marginal *R*^2^ = 0.322, conditional *R*^2^ = 0.619)				Outcome NVAI (Model marginal *R*^2^ = 0.410, conditional *R*^2^ = 0.835)		
	Beta	95% CI	*p*-value	Beta	95% CI	*p*-value
Different vitamin D treatments						
0	–	–		–	–	–
1	−1.1	−36, 34	0.949	−0.01	−0.15, 0.12	0.843
2	32	1.01, 64	**0.049**	−0.04	−0.17, 0.09	0.519
WC, cm	2.7	1.5, 3.9	**<0.001**	0.01	**0.01, 0.02**	**<0.001**
BMI, Kg/m^2^	−0.48	−2.8, 1.8	0.675	0.00	−0.01, 0.01	0.581

Values are expressed as unstandardized coefficients and 95% confidence intervals (CI). Abbreviations are the same as in Table 2; 0, without vitamin D supplementation; 1, cholecalciferol supplementation; 2, calcifediol supplementation. Bold numbers identify statistically significant associations.

## 4 Discussion

Our study aimed at evaluating whether different forms of vitamin D supplementation, when combined with a Mediterranean Hypocaloric Diet (MHD), could differentially affect vitamin D status, weight loss and visceral adiposity in individuals with obesity and vitamin D insufficiency or deficiency. The work emphasizes the potential benefits of vitamin D supplementation, especially in the form of calcifediol compared with cholecalciferol, when paired with an MHD. These benefits included the enhancement of plasma 25(OH)D concentration, as expected, but also the improvement of body composition, weight reduction and the decrease of visceral adiposity, described as the amelioration of visceral adiposity indices LAP and NVAI, in subjects with obesity and vitamin D insufficiency or deficiency.

Both cholecalciferol and calcifediol administration increased plasma 25(OH)D concentration in participants with obesity and vitamin D insufficiency, with calcifediol showing the greatest effect. All groups experienced significant reductions in body weight (BW), BMI, and waist circumference (WC) after following an MHD, confirming the efficacy of a low-calorie Mediterranean diet in promoting weight loss (25). However, the calcifediol supplemented group achieved the most substantial weight loss (−7.8 Kg) compared to those on MHD without vitamin D supplementation.

This finding aligns with previous research suggesting that calcifediol may be more effective than cholecalciferol in rapidly raising serum 25(OH)D concentration, particularly in individuals with obesity who may have altered vitamin D metabolism due to high adiposity (26–28), indicating a possible additive effect of calcifediol when combined with dietary interventions aimed at weight loss. These effects might be ascribed to a more rapid conversion of calcifediol to the active form of vitamin D compared to cholecalciferol, as well as to calcifediol’s ability to reduce meta-inflammation, that is the low-grade metabolic inflammation status and the chronic inflammatory response in obesity deriving from adipose tissue increased macrophage accumulation and release of adipokines, cytokines and chemokines, e.g., leptin, tumor necrosis factor (TNF-*α*), interleukins, and monocyte chemoattractant protein (MCP-1) (29). It has been reported that amelioration of vitamin D status in subjects with obesity and vitamin D deficiency, in combination with a hypocaloric diet, can lead to reductions in weight, fat mass and MCP-1 decrease.

Considering the relevance of meta-inflammation in promoting adiposopathy, and that visceral adipose tissue dysfunction is at the crossroad between chronic inflammation and metabolic disorders (30), our study therefore focused on Vitamin D supplementation effects on visceral adiposity. Our findings showed that, despite no significant differences in WC, FM and Body weight between the groups after 3 months of treatment, in the calcifediol-treated patients a significant improvement in the NVAI and LAP indices occurred, which are critical indicators of visceral fat and metabolic health. Of note, the computation of adiposity indices includes several parameters such as TG, HDL and MAP, highlighting the potential of calcifediol supplementation to mitigate the risks associated with visceral adiposity and obesity-related comorbidities. Furthermore, the potential of calcifediol supplementation to improve metabolic health and confer cardiovascular benefits by impacting visceral fat is confirmed by the reduction in diastolic blood pressure in the N group, independent of weight loss. This improvement underscores the importance of vitamin D supplementation in targeting visceral fat, which is a critical factor in metabolic health and cardiovascular risk. There is some evidence, although needing further understanding in large studies, suggesting that calcifediol supplementation in patients with severe obesity significantly increased 25(OH)D concentration in association with decreased inflammation and improvements in hypertension and dyslipidemia (31). Moreover, in cell lines and animal models, vitamin D supplementation has been reported to suppress cholesterol biosynthesis via vitamin D receptor-mediated pathways, suggesting a possible mechanism by which calcifediol could improve lipid profiles (32). In addition, in a pediatric population with obesity (33), vitamin D supplementation resulted beneficial for several lipid parameters. The lack of significant changes in visceral adiposity indices in the C and D groups may suggest that the effects of cholecalciferol on fat distribution are less pronounced compared to calcifediol. Moreover, the significant directional association between LAP and calcifediol treatment further supports the hypothesis that calcifediol may have a more favorable impact on visceral fat distribution compared to cholecalciferol. The association between LAP and WC indicates that visceral adiposity remains a critical factor in understanding the metabolic implications of vitamin D supplementation. This is in line with the concept that pre-vitamin D3 (cholecalciferol) showed greater liposolubility than 25(OH)D (calcifediol), and thus the use of calcifediol should be preferred in patients with obesity to avoid accumulation of the compound in fat depots (34).

Accordingly, it has been reported that the association between 25(OH)D and adiposity was stronger for visceral than subcutaneous abdominal adiposity, and that visceral adipose tissue is more strongly correlated with cardiovascular risks such as hypertension, hypertriglyceridemia, and the metabolic syndrome than the subcutaneous one (35). BMI is commonly used metric for assessing obesity; however, it does not differentiate between fat depots and lean mass, which may explain its lack of significant association with changes in LAP in our study. The significant association between waist circumference and NVAI, although not directly linked to vitamin D treatments, highlights the need for a comprehensive assessment of body fat distribution when evaluating the effects of dietary and supplemental interventions. The lack of association between NVAI and BMI emphasizes the need for a more detailed understanding of obesity, as BMI may not fully capture the complexities of body composition and its health implications. This finding underscores the importance of focusing on visceral fat rather than overall body weight or BMI in assessing health outcomes in individuals with obesity in the context of Vitamin D insufficiency. It has been recently reported in a longitudinal study that BMI does not impact on the elevation in 25(OH)D concentration after supplementation with calcifediol in young adults with vitamin D deficiency (36).

Interestingly, while both vitamin D supplementation groups (D and N) showed a reduction in fat mass percentage, only the N group exhibited improvements in fat-free mass, albumin levels, and calcium levels. This could indicate that calcifediol may promote not only weight loss but also healthier body composition during caloric restriction, potentially enhancing metabolic function linked to the role of vitamin D in protein metabolism and calcium homeostasis (37). Previous studies have shown that vitamin D might influence adipocyte function and adipose tissue metabolism (38), which could explain the favorable changes in body composition observed in our cohort of patients. Specifically, cholecalciferol and calcifediol modulate adipocyte physiology by inhibiting fat cell formation, promoting lipolysis, enhancing insulin sensitivity, and reducing inflammation and oxidative stress, primarily through AMPK, PPAR, and VDR-mediated mechanisms (39–43).

Our findings contribute to the growing body of literature addressing the complex interplay between vitamin D status, obesity, and metabolic health and provide insights into the role of vitamin D supplementation in the context of obesity management, particularly concerning visceral fat distribution. Moreover, it is worth noting that the bioequivalence between cholecalciferol 25,000 IU and 266 μg/month has not been clearly established. However, both regimens are able to achieve adequate serum vitamin D levels, although the relative difference in biological potency is estimated to range from 3- to 6-fold, with calcifediol being more effective than cholecalciferol in raising vitamin D concentrations (44). The calcifediol regimen used also follows the schedule suggested by the Italian Medicines Agency (note 96 of the Agenzia Italiana del Farmaco, AIFA, Rome, Italy) for vitamin D replenishment in cases of deficiency, with no associated safety concerns and comparable or even superior efficacy to long-term treatments with cholecalciferol (16). Nonetheless, it is essential to acknowledge some limitations of our study. The retrospective design limits the ability to establish causality, and the relatively small sample size, along with the exclusion of participants with some comorbidities, may affect the generalizability of the findings. Also, although regression models were adjusted for relevant confounders, the limited sample size did not allow for matching or balancing approaches, which restricts the ability to infer causal relationships. Future studies with larger cohorts and diverse populations are warranted to validate our data and explore long-term effects of vitamin D supplementation on body composition, visceral adiposity and metabolic health. Moreover, data in literature suggest that Vitamin D might play an anti-obesity role affecting the early adipogenesis (32); however, the specific local mechanisms through which vitamin D influences adiposity and adipocyte physiology require further investigation to deeply elucidate the effect of vitamin D metabolites on adipocyte function, inflammation, and insulin sensitivity.

Our study highlights the importance of monitoring vitamin D status in patients with obesity and assessing visceral adiposity in order to maximize the potential benefits of vitamin D supplementation. In particular, calcifediol, when combined with a Mediterranean hypocaloric diet, proved more effective than cholecalciferol in producing favorable effects on serum 25(OH)D concentrations, body composition, weight loss, and visceral adiposity in individuals with obesity, suggesting a meaningful role for this formulation in the metabolic management of obesity-related vitamin D deficiency. The improvements observed in metabolic parameters, the qualitative amelioration in body composition and visceral adiposity reduction, beyond weight loss, may represent key targets in obesity treatment strategies, further underscoring the relevance of incorporating vitamin D supplementation into weight management strategies to enhance overall health outcomes in the context of vitamin D insufficiency.

Supported by the literature, which shows that patients living with obesity have a higher prevalence of vitamin D deficiency compared to the general population, we recommend regular measurements of circulating vitamin D levels in this population so that dietary regimens can be supplemented appropriately to improve body composition and support weight control as part of comprehensive weight management programs.

Furthermore, considering additional factors, particularly the economic impact, there are no studies directly comparing the two forms of vitamin D that could guide the choice of one over the other. However, taking into account the costs of the two formulations in Italy, where calcifediol is less expensive than cholecalciferol, supplementation with the former could also be favored on this basis.

Overall, our results provide real-world evidence supporting the preferential use of calcifediol in combination with MHD in obesity-related vitamin D deficiency and encourage future prospective trials to confirm its long-term efficacy and metabolic benefits.

## Data Availability

The raw data supporting the conclusions of this article will be made available by the authors, without undue reservation.
